# Numerical Investigation of Longitudinal through Voids in Tunnel Secondary Lining Vaults and Steel Plate Strengthening

**DOI:** 10.3390/ma16124248

**Published:** 2023-06-08

**Authors:** Shuai Shao, Yimin Wu, Helin Fu, Sheng Feng, Jiawei Zhang

**Affiliations:** 1 School of Civil Engineering, Central South University, Changsha 410075, China; shaoshuai@csu.edu.cn (S.S.); fu.h.l@csu.edu.cn (H.F.); 2 Hubei Communications Planning and Design Institute Co., Ltd., Wuhan 430051, China; dabird@163.com; 3 Tianjin Municipal Engineering Design & Research Institute, Tianjin 300051, China; zhangjw.112358@gmail.com

**Keywords:** tunnel lining, longitudinal through void, numerical investigation, steel plate strengthening

## Abstract

This study investigates the influence of longitudinal through voids on vault lining. Firstly, a loading test was carried out on a local void model, and the CDP model was used for numerical verification. It was found that the damage to the lining caused by a longitudinal through void was primarily located at the void boundary. On the basis of these findings, an overall model of the vault’s through void was established using the CDP model. The effects of the void on the circumferential stress, vertical deformation, axial force, and bending moment of the lining surface were analyzed, and the damage characteristics of the vault’s through void lining were studied. The results indicated that the through void of the vault caused circumferential tensile stress on the lining surface of the void boundary, while the compressive stress of the vault increased significantly, resulting in a relatively uplifted vault. Furthermore, the axial force within the void range decreased, and the local positive bending moment at the void boundary increased significantly. The impact of the void increased gradually with the height of the void. If the height of the longitudinal through void is large, the inner surface of the lining at the void boundary will crack longitudinally, and the vault will be at risk of falling blocks or even being crushed.

## 1. Introduction

China has built a large number of highway tunnels in the past decade. By the end of 2019, China had built 19,067 highway tunnels. The total length of highway tunnels has reached 18966.6 km and is growing at a rate of 10% annually [1]. During the process of tunnel construction, the lining may develop a variety of defects or diseases due to various reasons [2,3,4,5,6]. One of the more common problems is voids on the vault [7].

The secondary lining is completed by pouring concrete onsite in the tunnel. The concrete is poured layer by layer, from bottom to top. However, challenges such as trapped gas within the formwork, limited concrete flowability, and excessive steel mesh density can give rise to empty spaces within the lining, particularly in the vault section [8]. The presence of voids causes a disconnection between the initial support and the secondary lining, resulting in an inability to distribute stress effectively. This directly impacts the stress conditions of the lining, preventing it from being uniformly loaded [9,10]. When there are voids in the lining, the stress concentration occurs near the edge of the voids, which increases the adverse tensile stress. If the void range is extensive and the void runs longitudinally through the lining, it can significantly impact the safety of the lining structure. If voids are not treated promptly, these voids may cause lining chipping, and even structural failure [11,12].

Numerical simulation is an effective method for studying engineering problems [13,14]. Numerous studies have shown that voids can have a significant effect on the internal forces of the lining [15,16,17,18]. Ding et al. [19] carried a 1:5 scaled-down model test to study the effect of voids on the lining. It was found that the void significantly reduced the bearing capacity of the lining, as well as increased the local deformation, and significant lining cracking showed at the void location. Zhang et al. [20] verified the effect of voids on lining in Ding’s test [19] by building a numerical model of CDP. The results showed that the bearing capacity of the lining decreased in an ‘s’-shaped curve with the increase in void. Through elastic–plastic finite element analysis, Wang et al. [21] found that voids can cause significant changes in the axial force and bending moment of the lining, and even lead to reversal of the bending moment, resulting in cracks or even failure of the lining. He et al. [22] made a void lining model on a 1:30 small scale, and they found that, when there is a void in the vault, the arch shoulder and arch knee are most likely to have compression damage, while the inner surface of the void lining is prone to cracking by tensile stress. Yasuda et al. [23] analyzed the effects of voids on the lining from the perspective of analytical calculations, and the results showed that voids could change the lining from being axial-force-dominated to bending-moment-dominated, resulting in the generation of adverse tensile stresses.

Research indicates that the addition of strengthening structures to the inner surface of the lining is an efficient solution to address the issue of vault voids. Currently, the techniques for addressing voids in the lining can be categorized into three groups: applying a high-strength fiber layer [24,25], affixing a steel plate [26,27,28,29,30], and constructing an umbrella arch [31,32]. Adding steel plates has significant advantages in dealing with lining void disease in operating tunnels. On the one hand, the strength of the steel plate is better than that of a fiber layer. On the other hand, it is faster and more convenient to install than an umbrella arch. The construction process has less impact on traffic and is not restricted by tunnel auxiliary facilities such as lights and fans.

In this research article, the impact of voids caused by insufficient thickness of the secondary lining was analyzed mainly through numerical simulation. The simulation was performed using the concrete damage plasticity (CDP) model by ABAQUS. Firstly, the local lining model with vault voids was made for the loading test, and the failure characteristics of the void lining were preliminarily analyzed. The corresponding CDP numerical model was established to verify the accuracy and reliability of the CDP constitutive model in simulating the void lining. Then, the whole lining model was established to study the impact on the vault through voids in the lining. On this basis, steel plate strengthening was used to treat the void model, verifying the effectiveness of steel plate strengthening in treating lining void disease.

## 2. Local Experiment and Numerical Verification

**Figure 1:**
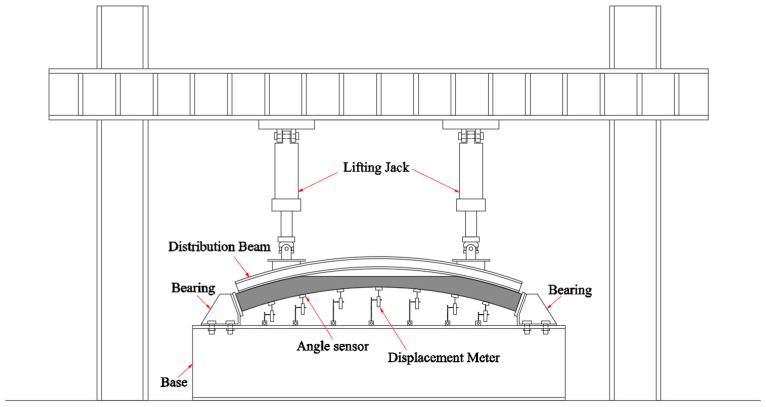
Layout of the loading system.

**Figure 2:**
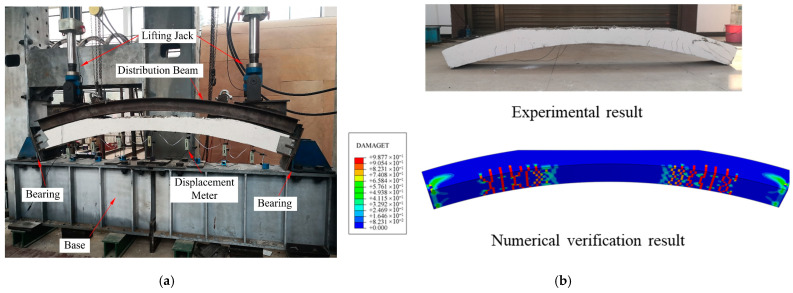
Experimental setup and results: (**a**) experiment device; (**b**) experimental result and numerical verification.

To explore the damage mechanism of voids, a static loading experiment was performed on the partially voided specimens of the vault. As shown in Figure 1, double hydraulic actuators were used for loading, and I-beams that were matching the curvature of the upper surface of the specimen were used for load distribution. The test revealed that the majority of the damage was localized around the boundary of the void (Figure 2). In order to verify these test results, a numerical model was established by the concrete damage plasticity model (CDP). The numerical results verified the test results, and they also proved the feasibility of the CDP simulation method in the study of void lining damage.

The aforementioned tests and numerical simulations indicate that the structural damage caused by voids primarily concentrated at the void boundary. However, the limitations of local specimens were evident: (1) the boundary of the local model was challenging to define accurately; (2) it was difficult to match the loading entirely with the actual situation, and loading symmetry was hard to guarantee; (3) partial support was prone to damage, ultimately affecting the loading. These issues impact the accuracy of the research results. To overcome these limitations, we established whole lining models with vault voids based on the CDP constitutive model to study the effect of voiding at different heights.

## 3. Numerical Model Setups

### 3.1. Geometry and FE Mesh

**Figure 3:**
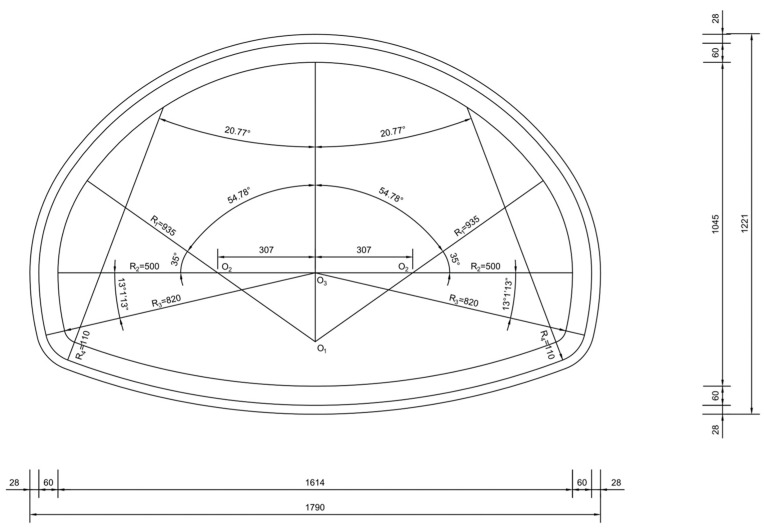
Lining dimensions.

**Figure 4:**
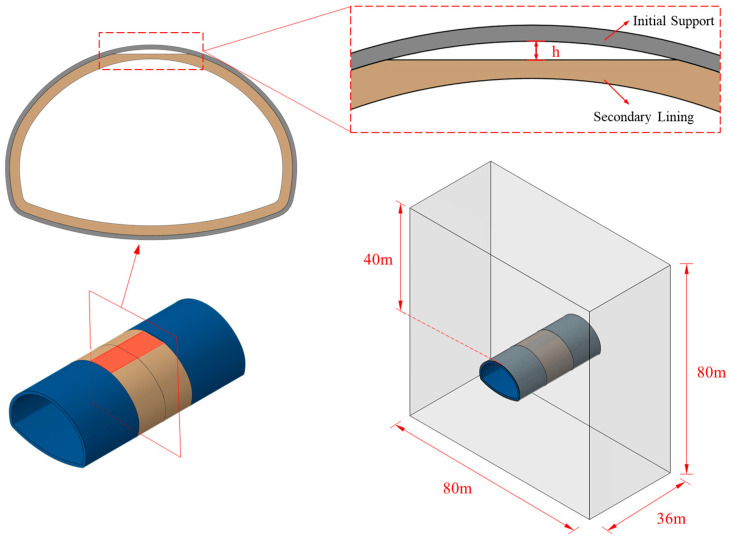
Model design.

Figure 3 displays the dimensions of the tunnel lining. The initial support was 28 cm, and the thickness of secondary lining H was 60 cm. The numerical model (Figure 4) was 80 m long, 80 m high, and 36 m thick, including three secondary lining pouring cycles. The secondary lining of the middle mold was a voided lining, while the front and rear linings were complete linings.

**Figure 5:**
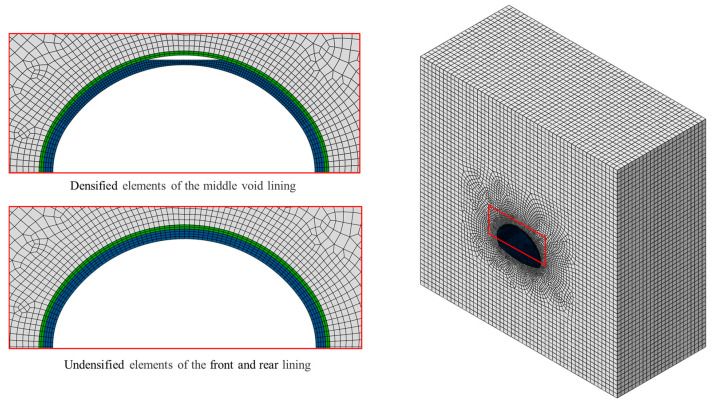
Element scheme.

As shown in Figure 5, the model was simulated by the C3D8R solid element. The initial support adopted two element layers, and the surface grid size was 15 cm × 14 cm. The secondary lining adopted four element layers; the surface grid size was 30 cm × 15 cm and the vault lining elements were densified within the 110° central angle. A sensitivity analysis was conducted on the lining units in the densified region. Four different sizes of lining surface units were considered: 10 cm × 15 cm, 15 cm × 15 cm, 30 cm × 15 cm, and 50 cm × 15 cm. The results revealed challenges in achieving convergence with a unit size of 10 cm × 15 cm, while a significant deviation in the computed results was observed with a unit size of 50 cm × 15 cm. To ensure both convergence and accuracy, a grid size of 15 cm × 15 cm was ultimately chosen for simulating the lining. The lining element nodes were aligned with the surrounding rock element nodes to make the calculation results more accurate.

There were five working conditions in this study, including one standard lining model and four kinds of void lining models. The heights of the lining void were set as H/4, H/2, 5H/8, and 3H/4, respectively. The detailed information of each working condition is shown in Table 1.

### 3.2. Material

The initial support adopted C25 concrete, and the secondary lining was made of C30 concrete. The elastic modulus was 3 × 10^4^ MPa. The Poisson’s ratio was 0.2, and the density was 2850 kg/m^3^. The detailed material parameters are shown in Table 2. The CDP model was used to simulate the upper half of the void lining, while the remainder of the lining was made of elastic material.

**Figure 6:**
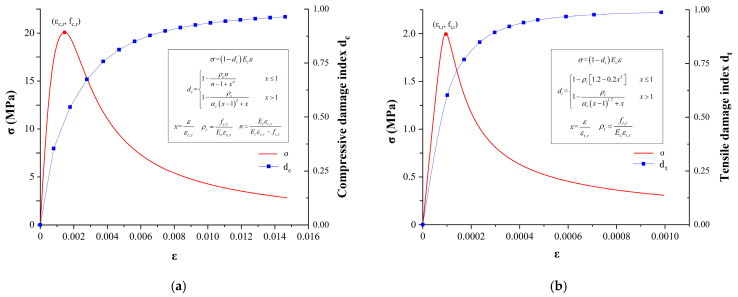
Stress–strain curve and damage factor for C30 concrete: (**a**) compression conditions; (**b**) tensile conditions.

The concrete damage plasticity (CDP) method is a constitutive model used to simulate the behavior of concrete under both plastic deformation and damage accumulation. It combines the concepts of plasticity and damage mechanics to capture the complex response of concrete under various loading conditions. The CDP method considers the evolution of plastic strains, damage variables, and their mutual interactions, providing a comprehensive framework to predict the structural response and failure of concrete structures. As shown in Figure 6, the plastic damage parameters used in this article were calculated using the following method: firstly, the stress–strain curve of concrete according to the Code for the Design of Concrete Structures (GB 50010-2010, 2015 Revised Edition) was obtained, and then the damage factor based on the parameter values in the stress–strain curve was calculated. The damage factor was calculated using the Najar damage theory [33].

### 3.3. Loading and Boundary Conditions

The bottom surface of the model was fixed, and the normal displacement was limited around it. The load depth was 100 m in total and consisted of two parts; one part was buried at a depth of 40 m above the lining, and the other part had a uniform surface load of 1.44 MPa applied on the top surface, which was equivalent to a depth of 60 m.

## 4. Results

### 4.1. Circumferential Stress on the Lower Surface

**Figure 7:**
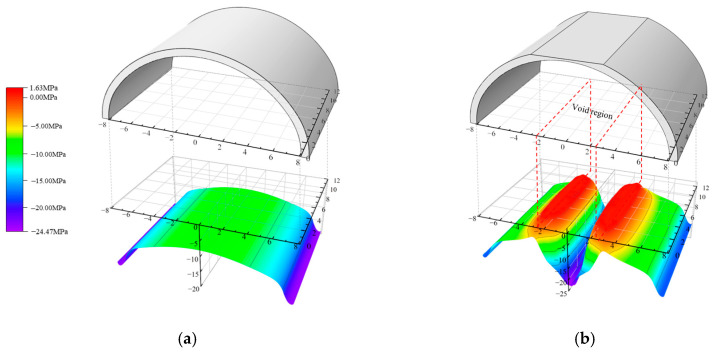
Circumferential stress on the lower surface: (**a**) V0; (**b**) V(3/4).

Figure 7a,b show the circumferential stress on the lower surface of the lining when there were no voids and a 3H/4-high void, respectively. The lining without the void was in a state of compression as a whole, while the stress state of the void lining changed significantly. Voids led to a tensile zone at the void boundary on the lower surface of the lining, representing an adverse force state. It can be seen that the critical section was at the position of 6 m in the longitudinal direction.

**Figure 8:**
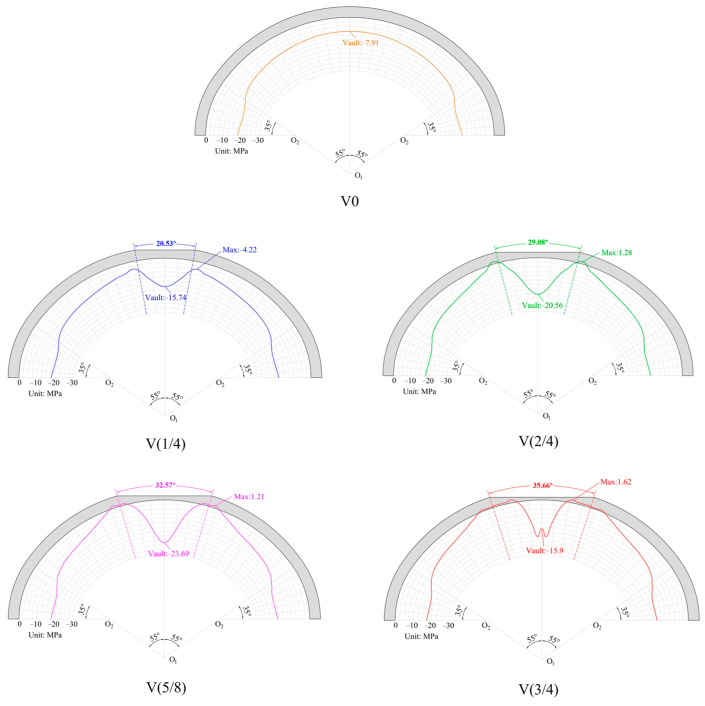
Circumferential stress of the critical sections.

The circumferential stress of the critical sections under each working condition is shown in Figure 8. From the figure, it is evident that the lining without the void was in a state of compression, and it was relatively uniform. The compressive stress within the vault section was 7.91 MPa. However, voids caused significant changes in the circumferential stress. When the void height was H/4, the compressive stress at the void boundary decreased to 4.22 MPa, and there was a trend toward tensile stress. The compressive stress within the vault section was 15.47 MPa, which increased by 95.57% compared with the lining without a void. When the void height was H/2, tensile stress occurred at the void boundary. The circumferential span of the tensile stress was 119.46 cm, and the maximum tensile stress was 1.277 MPa, but it did not reach the concrete tensile strength. The compressive stress of the vault further increased to 20.56 MPa, which was an increase of 159.92% compared with the lining without a void. When the void height was expanded to 5H/8, the maximum circumferential tensile stress was 1.214 MPa, and the circumferential tensile stress span expanded to 213.3 cm. The compressive stress of the vault further increased to 23.69 MPa, which was an increase of 199.49% compared with the lining without a void. In contrast with the H/2 void height, the maximum circumferential tensile stress of the void at 5H/8 did not improve much. This indicates that the compressive stress exceeded the compressive strength here, and that the concrete stiffness degraded. Similarly, when the void height was 2 H/3, the maximum tensile stress at the void boundary was 1.62 MPa, and the range of the tensile stress further increased to 444.18 cm. The compressive stress at the vault reduced to 15.9 MPa, indicating that the vault stress exceeded the compressive strength, and the stiffness degraded.

### 4.2. Vertical Deformation

**Figure 9:**
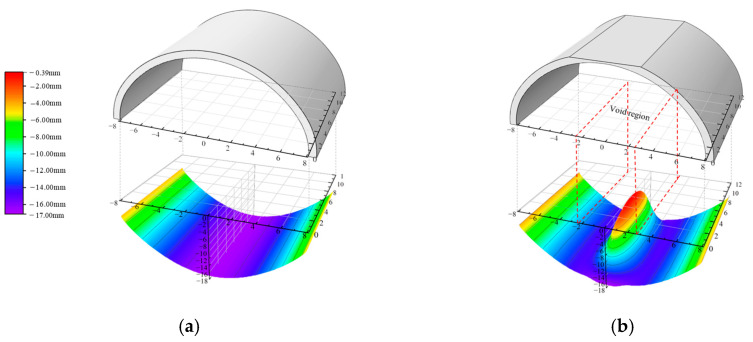
Vertical deformation: (**a**) V0; (**b**) V(3/4).

Figure 9a,b show the vertical deformation of the lower surface when there was no void and when the void height was 3H/4, respectively. As depicted in the figure, the influence of the void on the vertical deformation mainly concentrated in the void range, which manifested as an upward uplift of the lining. Moreover, constrained by the adjacent two mold linings, the void lining also showed characteristics of being high in the middle and low on both sides in the longitudinal direction. The critical sections were at the position of 6 m in the longitudinal and circumferential directions.

**Figure 10:**
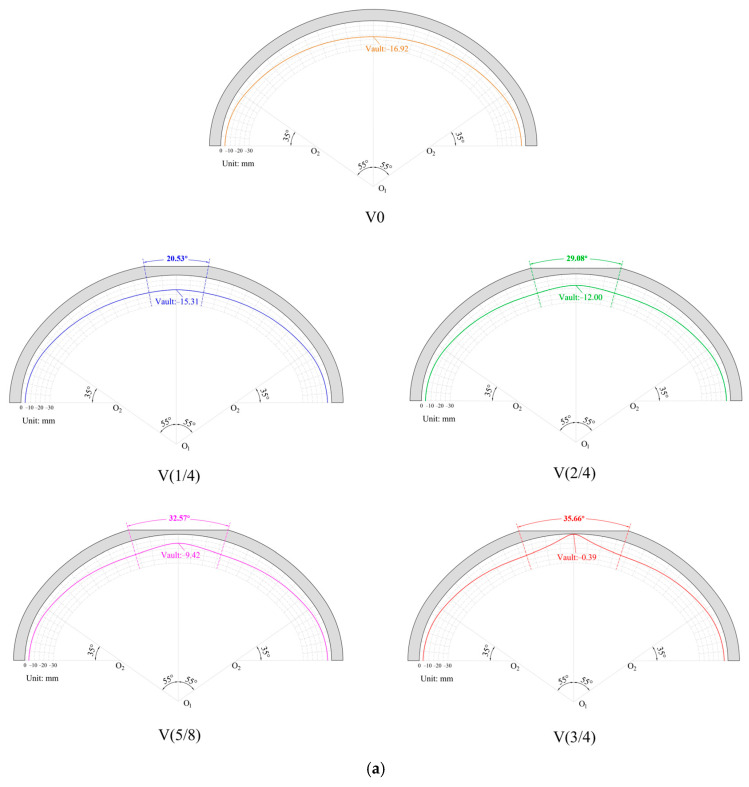

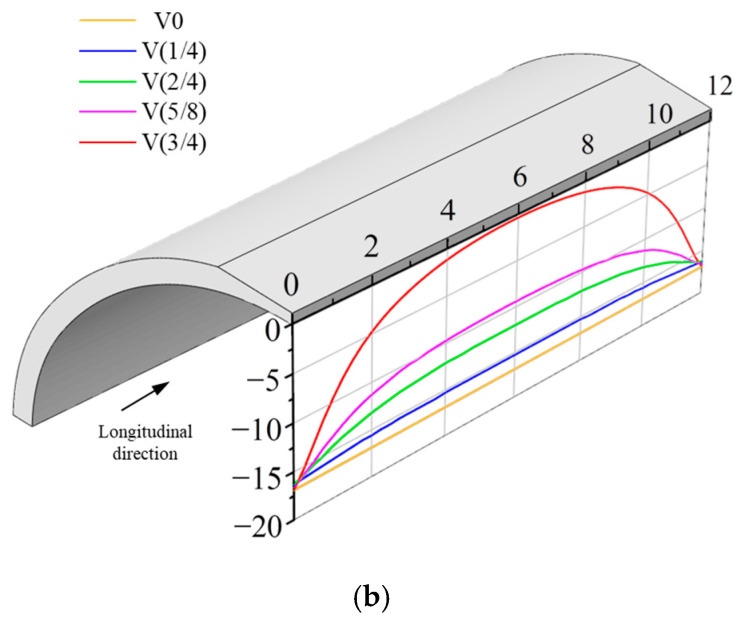
Vertical deformation of critical section: (**a**) circumferential critical section; (**b**) longitudinal critical section.

To analyze the variations in the vertical deformation under different void heights, the illustrations in Figure 10a,b demonstrate the vertical deformation of the critical sections. The vault area was identified as the location from the circumferential perspective where the presence of the void had the most significant impact on the vertical deformation. When there was no void in the lining, the vertical deformation of the vault was −16.9 mm. When there was a void with a height of H/4, the vertical deformation of the vault changed to −15.3 mm, which was 1.6 mm higher than that of the non-void lining. As the height of the void increased, there was a gradual rise in the relative uplift observed at the vault position. When the void height was 3H/4, the vault bulge was the most obvious, and the vertical deformation of the vault was only −0.39 mm, which was 16.51 mm higher than that without a void. From the longitudinal view of the vault, the vertical deformation showed the characteristics of being “high in the middle and low on both sides”. The vertical deformation near the construction joint was hardly affected by the void, but the bulge in the middle increased with the increase in the void.

### 4.3. Axial Force

**Figure 11:**
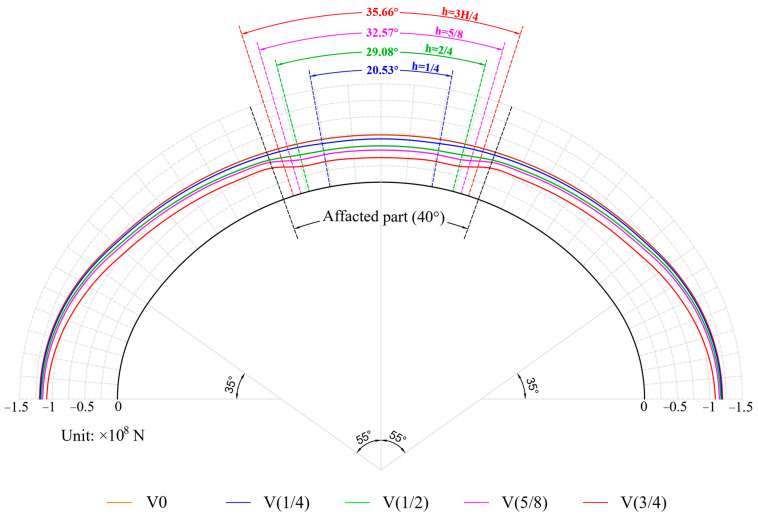
Axial force.

**Figure 12:**
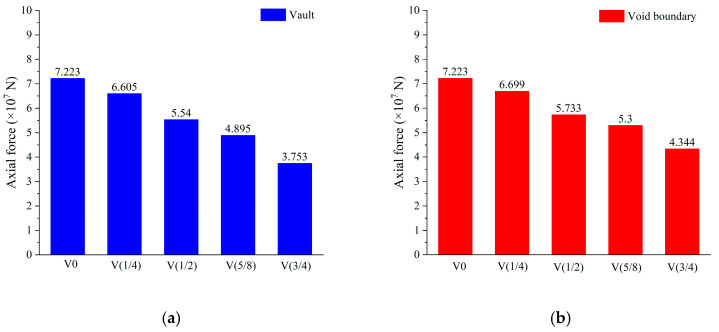
Vertical deformation at special positions: (**a**) vault; (**b**) void boundary.

Figure 11 depicts the axial force variation curve of the upper half arch of the void lining, with the negative value representing compression. On the whole, the voids led to a decrease in axial force, and the decrease range increased as the height of the void increased. The axial force in the void range was less than that in the nonvoid part, which was more obvious when the void height was large. Figure 12a,b show the axial force at the vault and at the void boundary, respectively. The figure illustrates a gradual decrease in the axial force with an increase in void height. At the vault position, the axial force was 7.223 × 10^8^ N with no void, and the axial force decreased by 8.56% and 23.3% when the void heights were H/4 and H/2, respectively. When the void height increased to 5H/8 and 2H/3, the axial force at vault reduced by 32.23% and 48%, respectively, compared with that without the void.

### 4.4. Bending Moment

**Figure 13:**
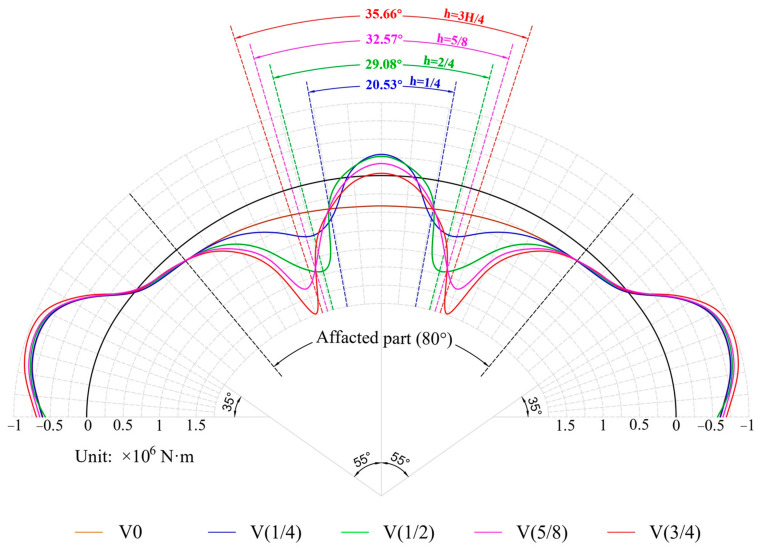
Bending moment.

**Figure 14:**
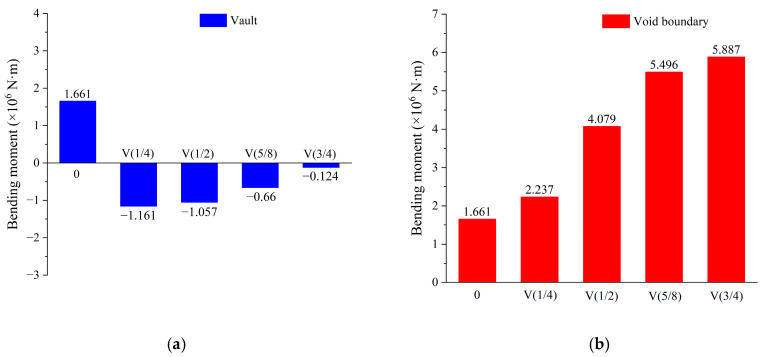
Bending moment at special positions: (**a**) vault; (**b**) void boundary.

Figure 13 shows the distribution of the bending moment along the circumferential critical section, with the tension on the lower surface being positive. As can be seen from the figure, the voids caused significant changes in the bending moment distribution, but the influence was mainly concentrated in the 80° range on both sides of the vault. In the absence of voids, the positive bending moment at the vault reached its maximum and gradually diminished toward both sides. Once the void occurred, the bending moment at the vault decreased rapidly and turned to the upper side tension. At the same time, the bending moment at the void boundary also increased rapidly. Figure 14 depicts the bending moment at the vault and void boundary. Only when there was no void was the bending moment of the vault positive; that is, the lower part was in tension. When the lining was voided, the bending moment at the vault became negative; that is, the lower part was compressed and the upper part was tensioned, which also corresponded to the compression damage on the lower surface of the vault. At the void boundary, the bending moments were positive; that is, the lower surface was in tension. With the increase in the void height, the bending moment gradually increased from 1.661 × 10^6^ N·m to 5.887 × 10^6^ N·m, which also corresponded to the tensile damage on the lower surface of the void boundary.

### 4.5. Damage

**Figure 15:**
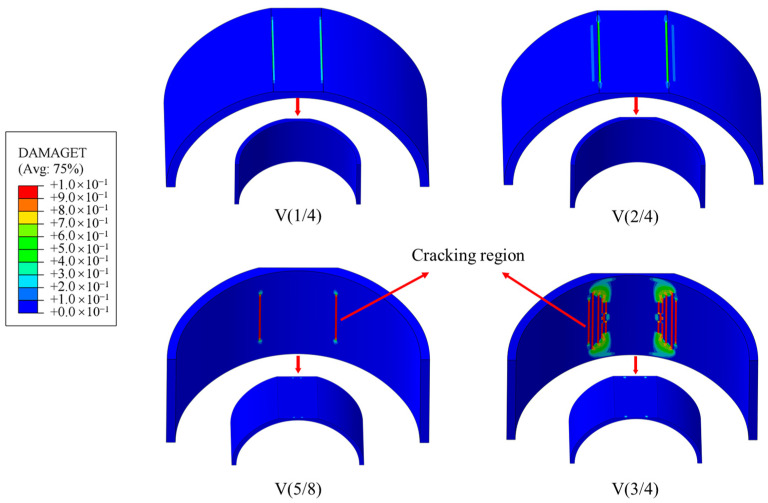
Tensile damage.

Figure 15 shows the results from tensile damage of the lining under various void heights. The lining without any voids had no obvious damage and is not listed. When there was a void in the vault, the damage showed a certain law with the increase in void height. When the void height was H/4 and H/2, the tensile damage of the secondary lining concentrated at the void boundary of the upper surface, the damage degree was small, and the length was short. When the void height increased to 5H/8, the damage on the upper surface of the secondary lining disappeared, and obvious cracking damage began to appear on the lower surface. It developed longitudinally along the void boundary, but the range was small, and there was only one along the left and right boundaries. When the void height continued to increase to 3H/4, the number of longitudinal cracks on the lower surface increased significantly, and each void boundary increased to five cracks, which indicated that the range of the tensile zone increased significantly, and the degree of damage intensified. However, at the position close to the construction joint, the tensile damage was limited due to the restraint of the front and rear lining.

**Figure 16:**
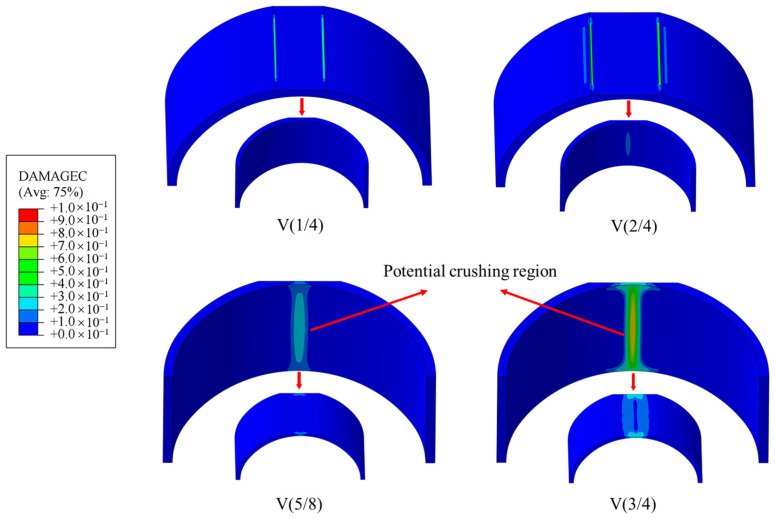
Compression damage.

In terms of compression damage, as shown in Figure 16, when the height of the void was H/4, noticeable compression damage was observed on the outer surface of both sides of the void boundary, but the damage value was only 0.11, which is small. When the void height was H/2, the damage degree at the void boundary of the outer surface expanded to about 0.17, and damage began to occur at the midspan of the lower surface. As the void height continued to increase, the compression damage state of the lining changed significantly. Compressive damage on the inner surface primarily occurred under conditions with larger voids. When the void height reached 5H/8, the central region of the inner surface exhibited damaged areas, with a maximum damage value of 0.26. However, when the void height reached 3H/4, the maximum damage value at the central position of the inner surface increased to 0.84, indicating significant compressive damage in the lining at the vault region. This suggests the potential risk of concrete spalling or even structural collapse.

## 5. Steel Plate Strengthening Model for Void Lining

Steel plate strengthening is a common method used to treat void defects of linings. This strengthening approach involves utilizing chemical anchor bolts and epoxy adhesive to stick the steel plate onto the inner surface of the void lining. The steel plate and the lining work cooperatively to reduce the stress concentration due to voids, which limits the development of lining damage. On the basis of the simulation of the void lining, a simulation verification of steel plate strengthening was carried out under the working condition of a void height of 3H/4. The mechanism of steel plate strengthening was analyzed, which provided a basis for the design of a steel plate strengthening scheme.

### 5.1. Simulation of the Steel Plates Strengthening to Void Lining

**Figure 17:**
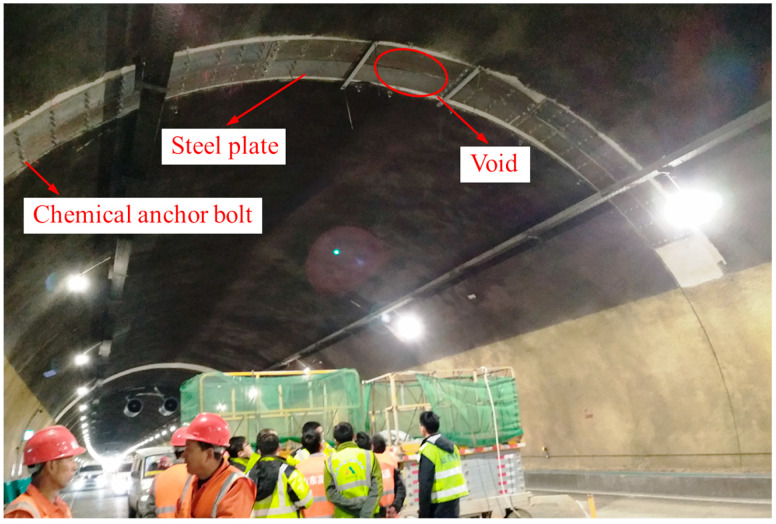
Steel plate strengthening site.

Figure 17 shows the steel plate strengthening scheme of a tunnel. According to the simulation of the void lining, the stress concentration caused by the void was near the void boundary, and there was no large stress located far from the void boundary. Therefore, the void and the vicinity of the void boundary were the main positions that were to be strengthened.

**Figure 18:**
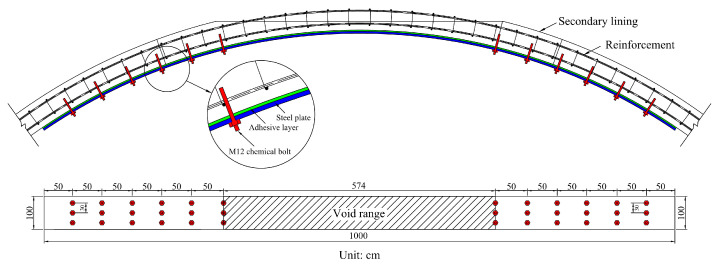
Steel plate strengthening scheme.

According to the simulated stress concentration area, Figure 18 illustrates the scheme for steel plate strengthening. The steel plate was 10 m long, 1 m wide, and 1 cm thick. The steel plate and the lining surface were bonded with epoxy adhesive and reinforced with M24 chemical anchor bolts.

**Figure 19:**
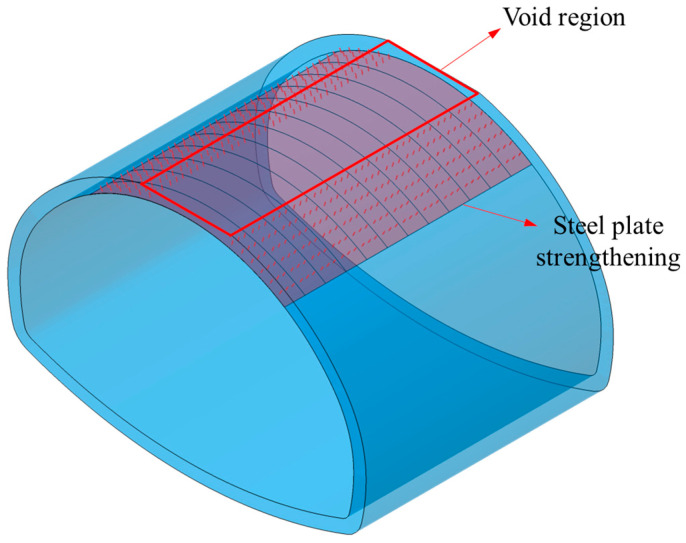
Model setup.

The steel plate strengthening scheme was applied to the void 3H/4 lining to study its strengthening effect. In the simulation model shown in Figure 19, the M24 chemical anchor bolts were simulated by beam element, with a circular section and a diameter of 24 mm. The bolt elements were embedded into the interior of the lining elements through embedded region constraints; the steel plate was simulated with a shell unit with the unit thickness set to 10 mm. The steel adopted a bilinear constitutive law. The elastic modulus was 210 GPa, and the yield stress was 235 Mpa. The contact between the steel plate and the lining was set to cohesive contact. The normal strength was 30 Mpa, the shear strength was 20 Mpa, and the total plastic deformation was 0.008 mm.

### 5.2. Evaluation of Strengthening Effect

**Figure 20:**
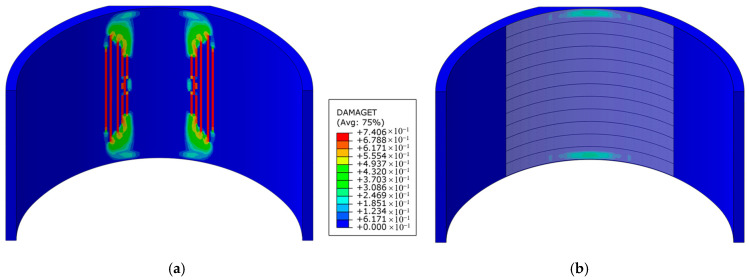
Damage comparison of steel plate strengthening: (**a**) unstrengthened lining; (**b**) strengthened lining.

Figure 20 shows the comparison between the damage of the strengthened lining and the unstrengthened model. It can be seen from the figure that, after steel plate strengthening was applied, tensile damage at the void boundary was completely eliminated. These findings indicate that implementing steel plate reinforcement could effectively mitigate damage to the lining resulting from voids.

**Figure 21:**
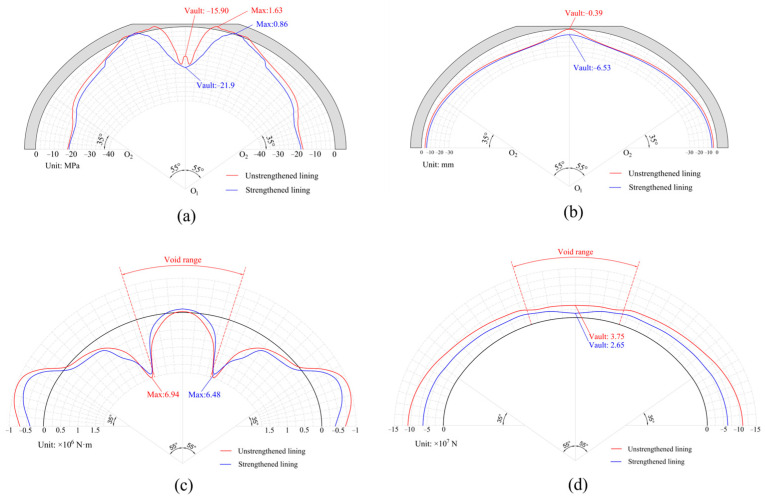
Comparison of internal force of strengthened and unstrengthened void lining: (**a**) circumferential stress; (**b**) vertical deformation; (**c**) bending moment; (**d**) axial force.

The internal force and deformation of the lining model strengthened with steel plates are shown in Figure 21. The maximum circumferential stress on the inner surface of the strengthened lining was 0.86 MPa, which was 47.24% lower compared to the unreinforced lining. The range in positive stresses at the void boundary of the strengthened lining significantly reduced from 2.22 m to 0.614 m, indicating that the tensile damage at this location was effectively improved. The concrete at the vault was obviously compressed, and the vault lining of the unstrengthened model yielded and showed compression damage; however, there was no damage to the vault of the strengthened lining, and the concrete was not plastic yielding. The vault’s vertical deformation in the strengthened lining was reduced from 0.39 mm to 6.53 mm, and the deformation difference between the vault and the void boundary reduced from 14.11 mm to 8.58 mm. The overall deformation of the strengthened lining was more coordinated. These findings indicate that employing steel plate strengthening can effectively mitigate uplift of the vault caused by the presence of voids. Due to the fact that the steel plate did not significantly change the cross-sectional size, there was no significant difference in the bending moment between the strengthened and unstrengthened linings. The maximum bending moment at the degassing boundary was reduced from 6.94× 10^6^ N·m to 6.48× 10^6^ N·m, a reduction of only 6.63%. However, the axial force of the void lining strengthened with steel plates decreased overall, with the axial force at the vault decreasing from 3.75 × 10^7^ N to 2.65 × 10^7^ N.

## 6. Conclusions

In this study, the damage characteristics of voids were explored through local model tests, and the numerical model of a test block was established to verify the effectiveness and reliability of the CDP constitutive model in simulating lining voids. On this basis, the whole lining model that used the CDP constitutive model was established. The specific conclusions are as follows:

1. The impact of the through void on the lining stress was mainly concentrated in the lining of the void part and at the void boundary. This shows that the tensile stress range and cracks of the vault through void were concentrated on the void boundary, mainly along the longitudinal crack; moreover, the number of cracks gradually increased with increasing void height. The damage caused by compressive stress was mainly concentrated in the vault, which may carry the risk of falling blocks or even crushing.
2. The influence of the through void on the vertical deformation of the lining was mainly manifested in the relative bulge of the lining inside the void zone. With the progressive increase in void height, the midspan bulge value increased gradually. The vertical deformation change caused by the void mainly concentrated in the local range of 40° of the vault. Longitudinally, constrained by the front and rear linings, the vault bulge was maximum at the middle of the lining, and gradually diminished toward both sides; there was no bulge at the construction joint.
3. Through voids led to decreasing lining axial force. A greater void height led to a greater reduction in the axial force. At the vault, the axial force of the lining without a void was 7.223 × 10^8^ N, while the axial force of the 3H/4 void lining decreased to 3.753 × 10^8^ N, which was a 48% reduction. At the same void height, the influence of through void on the axial force mainly concentrated in the void range, and the axial force of the lining in the void part was significantly lower than that in the non-void part.
4. The influence of through void on the bending moment was large, mainly concentrated in the range of 80° of the arch crown. When the void occurred, the positive bending moment at the void boundary increased significantly, which led to greater tensile stress on the lower surface. The vault bending moment became negative, indicating that the lower surface of the vault was under compression.
5. The tensile stress on the lower surface of the steel plate strengthening void lining significantly decreased in the reinforcement area. The maximum circumferential tensile stress at the void boundary reduced from 1.63 MPa to 0.86 MPa, which was 47.24% less than the unstrengthened void lining. The compressive yielding and damage of the vault disappeared. The uncoordinated deformation at the vault was also improved, and the deformation difference between the vault and the void boundary was reduced from 14.11 mm to 8.58 mm, making the lining deformation more uniform.

## Figures and Tables

**Table 1:** Detailed information of the specimens.

Type	Code Name	Void Depth h
Standard lining	V0	N/A
Void lining	V(1/4)	H/4
	V(1/2)	H/2
	V(5/8)	5H/8
	V(3/4)	3H/4

**Table 2:** Detailed information of the material parameters.

	Density (kg·m^−3^)	Elastic Modulus (GPa)	Poisson’s Ratio	Internal Friction Angle (°)	Cohesion (kPa)
Surrounding rock	2400	1.5	0.3	30	700
Initial support	2400	28	0.2		
Secondary lining	2850	30	0.2		

## Data Availability

All data are available from the authors.
